# Supplementary material for: Effectiveness of Gabapentin as a Benzodiazepine-Sparing Agent in Alcohol Withdrawal Syndrome
Source: Medicina (Kaunas). 2024 Jun 19;60(6):1004. doi: 10.3390/medicina60061004 (PMC11205746; doi:10.3390/medicina60061004)
Supplement: Supplementary file 1 [file medicina-60-01004-s001.zip › CIWA-Supplementary file.pdf]

This sheet shows our local alcohol withdrawal protocol orders using the clinical institute withdrawal assessment of alcohol scale, revised (CIWA-Ar)

All patients admitted with suspected or confirmed alcohol withdrawal are to receive:

- ☐ By Mouth (PO) for 3 days
  - Thiamine 100 mg by mouth daily, first dose at the time of diagnosis
  - Folic Acid 1 mg by mouth daily, first dose at the time of diagnosis
  - Multivitamin 1 tablet by mouth daily, first dose at the time of diagnosis

Recurrent CIWA-Ar score calculation, frequency as ordered by the treating physician.

- ☐ Any CIWA-Ar score of 8 or above: give the withdrawal medication ordered, document the score in the electronic medical record, repeat and reassess CIWA-Ar score and O_2 saturation within 1 hour after withdrawal medication administration.

Laboratory: Perform upon initiation of CIWA Protocol, then as needed

□ Renal Panel □ Magnesium □ CBC with auto differential □ Urine Drug Screen □ Liver function test □ Amylase □ Lipase □ PT/aPTT □ INR □ Blood Alcohol Level

Nursing Care:

Symptom-triggered assessment is based on the CIWA-Ar scoring and sedation is based on the Richmond Agitation and Sedation Scale (RASS)

Upon admission, we evaluate the patient's individual score on the CIWA-Ar scale and the RASS assessment scale

The nurse is instructed to use the CIWA-Ar Monitoring Flow Sheet and document CIWA-Ar score.

- Once the medication is administered following baseline CIWA-Ar score, reassess within one hour, then follow the procedure below:
  - If the CIWA-Ar score is <8
    - Reassess every 4 hours
  - If the CIWA-Ar score is 8-18
    - Reassess every 2 hours
  - If the CIWA-Ar score is >18
    - Reassess every 1 hour

Use the Richmond Agitation and Sedation Scale (RASS) Assessment.

- Following baseline RASS score, reassess at least every 2 hours
- If RASS is -3 or below
  - Hold one dose of benzodiazepine, then reassess.
- If RASS is ≥ -3 proceed to CAM-ICU assessment

Use the CAM-ICU Assessment for detecting and monitoring the development of hallucinations and delirium.

- If CAM-ICU is negative: no delirium
- If CAM-ICU is positive: delirium is present

Nurses are instructed to notify the physician if:

- Unable to arouse the patient
- O_2 saturation is below 92% and the patient is on 2 L O_2
- CIWA-Ar score is above 15
- RASS score is -3 or below
- Seizure activity
- New-onset delirium and/or hallucinations
- If Vitals show: SBP < 90 mmHg; DBP < 60; HR <60 bpm; RR < 12 **Hold benzodiazepine in this case**

Alcohol Withdrawal Management:

Benzodiazepines:

For a CIWA-Ar score below 8, no treatment is recommended

Nurses are instructed to hold benzodiazepines for SBP < 90 mmHg, DBP < 60 mmHg, HR < 60 bpm, or RR < 12

- ☐ **Symptom-triggered Schedule:**
  - ☐ Preferred Regimen ****Caution for renal dysfunction****
    - Chlordiazepoxide 50 mg by mouth every 4 hours (q4h) as needed for CIWA-Ar score of 8 or above for 24 hours followed by 50 mg by mouth every 6 hours as needed for CIWA-Ar score of 8 or above for 24 hours, then 25 mg by mouth q8h as needed for CIWA-Ar score of 8 or above for 24 hours then 25 mg by mouth q12h as needed for CIWA-Ar score of 8 or above for 24 hours then 25 mg by mouth at bedtime as needed for CIWA-Ar score of 8 or above for 24 hours.
    - Diazepam 10 mg by mouth every 1 hour as needed for CIWA-Ar score of 8 or above for 72 hours
  - ☐ Alternative Regimen: ****Recommended in elderly and liver dysfunction patients****
    - Lorazepam 2 mg by mouth every 1 hour as needed for CIWA-Ar score of 8 or above for 72 hours
  - ☐ NPO patients
    - Lorazepam (Ativan) 2 mg IV every 1 hour as needed for CIWA-Ar score of 8 or above for 72 hours
- ☐ **Fixed-Dose with PRN Schedule: **Recommended for patients with a previous history of ETOH withdrawal seizures, delirium tremens, cardiopulmonary disease, surgical, or pregnant patients or patients with CIWA-Ar > 18**
  - ☐ Preferred Regimen ****Caution for renal dysfunction****
    - Diazepam 10 mg by mouth every 6 hours for 8 doses, then 5 mg by mouth every 6 hours for 8 doses
    - Plus, Diazepam 5 mg by mouth every 1 hour as needed for CIWA-Ar score of 8 or above, discontinue after 4 days
  - ☐ Alternative Regimen ****Recommended in elderly and liver dysfunction patients****

- Lorazepam 2 mg by mouth every 6 hours for 8 doses, then 1 mg by mouth every 6 hours for 8 doses
    - Plus, Lorazepam 1 mg by mouth every 1 hour as needed for CIWA-Ar score of 8 or above, discontinue after 4 days
- ☐ Chlordiazepoxide (Librium)
    - Chlordiazepoxide (Librium) 50 mg q1h for 3 doses then 50 mg by mouth q4h for 24 hours followed by 50 mg by mouth q6h then 25 mg by mouth q8h x 24 hours then 25 mg by mouth q12h x 24 hours then 25 mg by mouth at bedtime x 24 hours
    - Plus, Chlordiazepoxide 50 mg q4h as needed for CIWA-Ar score of 8 or above, discontinue after 4 days

☐ **NPO patients**

- Lorazepam (Ativan) 2 mg IV every 6 hours for 8 doses, then 1 mg IV every 6 hours for 8 doses
- Plus, Lorazepam (Ativan) 1 mg IV every 1 hour as needed for CIWA-Ar score of 8 or above, DC after 4 days

In patients with contraindications to benzodiazepines or as an adjunct to benzodiazepines:

☐ **Gabapentin**

- Gabapentin 1200 mg PO for 1 dose followed by 600 mg PO q6h for 4 doses then 200 mg PO three times daily (TID) for 3 days followed by 200 mg PO twice daily (BID) for 3 days.

* **Visual Disturbances** – ask "Does the light appear to be too bright? Is its color different? Does it hurt your eyes? Are you seeing anything that is disturbing you? Are you seeing things you know are not there?"

** **Tactile Disturbances** – ask "Do you have any itching, pins & needles sensations, burning or numbness or do you feel like bugs are crawling under your skin?"

*** **Auditory Disturbances** – ask "Are you more aware of sounds around you? Are they harsh? Do they frighten you? Are you hearing anything that is disturbing you? Are you hearing things you know are not there?"

CIWA scoring documentation

**Date ⇨**																	
**Time ⇨**																	
**Initials ⇨**																	
Nausea and vomiting																	
Paroxysmal sweats																	
Agitation																	
Headache																	
Anxiety																	
Tremor																	
Visual disturbances																	
Tactile disturbances																	
Auditory disturbances																	
Orientation																	
**Total Score *(max = 67)***																	
Drug ____________ Dose given:																	
O_2 Saturation																	

RN Signature ____________ Date ____________ RN Signature ____________ Date ____________

RN Signature ____________ Date ____________ RN Signature ____________ Date ____________

Table S1. Generalized linear model for length of hospital stay.

Effect	Num DF	Den DF	F Value	Pr > F
Medication Group	1	80.4	0.95	0.3338
Age	1	4348	2.47	0.1163
Sex	1	4354	2.81	0.0938
Race	1	4352	0.01	0.9296
BMI	1	4348	8.16	0.0043
CKD	1	4344	0.14	0.7112
Liver Disease	1	4353	0.01	0.9261
GAD	1	4354	36.50	<.0001
ELIX	1	4352	114.47	<.0001

BMI: body mass index, CKD: chronic kidney disease, GAD: generalized anxiety disorder, ELIX: Elixhauser comorbidity index.

Table S2. Generalized linear model of length of ICU stay.

Effect	Num DF	Den DF	F Value	Pr > F
Medication Group	1	9.16	0.57	0.4687
Age	1	423	4.24	0.0401
Sex	1	423	4.09	0.0438
Race	1	423	0.74	0.3911
BMI	1	417	0.25	0.6147
CKD	1	416	0.29	0.5896
Liver Disease	1	422	1.05	0.3051
GAD	1	421	0.11	0.7450
ELIX	1	424	25.28	<.0001

BMI: body mass index, CKD: chronic kidney disease, GAD: generalized anxiety disorder, ELIX: Elixhauser comorbidity index.
